# Development and application of a TaqMan single nucleotide polymorphism genotyping assay to study infectious laryngotracheitis virus recombination in the natural host

**DOI:** 10.1371/journal.pone.0174590

**Published:** 2017-03-28

**Authors:** Carlos A. Loncoman, Carol A. Hartley, Mauricio J. C. Coppo, Paola K. Vaz, Andrés Diaz-Méndez, Glenn F. Browning, Sang-won Lee, Joanne M. Devlin

**Affiliations:** Asia Pacific Centre for Animal Health, Faculty of Veterinary and Agricultural Sciences, The University of Melbourne, Victoria, Australia; Mathematical Institute, HUNGARY

## Abstract

To date, recombination between different strains of the avian alphaherpesvirus infectious laryngotracheitis virus (ILTV) has only been detected in field samples using full genome sequencing and sequence analysis. These previous studies have revealed that natural recombination is widespread in ILTV and have demonstrated that recombination between two attenuated ILTV vaccine strains generated highly virulent viruses that produced widespread disease within poultry flocks in Australia. In order to better understand ILTV recombination, this study developed a TaqMan single nucleotide polymorphism (SNP) genotyping assay to detect recombination between two field strains of ILTV (CSW-1 and V1-99 ILTV) under experimental conditions. Following *in vivo* co-inoculation of these two ILTV strains in specific pathogen free (SPF) chickens, recovered viruses were plaque purified and subjected to the SNP genotyping assay. This assay revealed ILTV recombinants in all co-inoculated chickens. In total 64/87 (74%) of the recovered viruses were recombinants and 23 different recombination patterns were detected, with some of them occurring more frequently than others. The results from this study demonstrate that the TaqMan SNP genotyping assay is a useful tool to study recombination in ILTV and also show that recombination occurs frequently during experimental co-infection with ILTV in SPF chickens. This tool, when used to assess ILTV recombination in the natural host, has the potential to greatly contribute to our understanding of alphaherpesvirus recombination.

## Introduction

Infectious laryngotracheitis virus (ILTV) causes mild to severe respiratory tract disease in chickens that results in major economic losses as a consequence of increase mortality and decreased weight gain and egg production in poultry industries worldwide [1]. In 2012, our laboratory reported that new dominant field strains, classified as class 8 and class 9 ILTV, using the Australian ILTV PCR-RFLP genotyping system [2] arose due to natural recombination between two ILTV vaccine strains in Australia [3]. These new field strains have been responsible for widespread outbreaks of disease and also have displaced previously dominant field strains in some regions in Australia [3–5]. More recently, another virulent recombinant virus, class 10 ILTV, has emerged in Australia to become dominant in some poultry producing regions [6]. Recently a review compiling ten years of research has helped to understand the importance of recombination in alphaherpesviruses evolution [7], showing that recombination allows some alphaherpesviruses to persist, evolve, and become more virulent through time [3, 5, 8–12]. As alphaherpesviruses have a DNA polymerase with a highly efficient proofreading activity, these viruses have very low genetic mutation rates [13, 14] and so recombination can be particularly important for herpesvirus genome diversification [3, 15, 16].

Although ILTV recombination has been detected and studied in field conditions through full genome sequencing and sequence analysis of recovered isolates [3, 5, 6, 17], genome recombination of these viruses has not been studied under experimental conditions. In this study we aimed to develop a rapid and simple TaqMan SNP genotyping assay, as an alternative to full genome sequencing and sequence analysis, to detect ILTV recombinants and to study ILTV recombination under experimental conditions in the natural host.

## Material and methods

### Viruses

V1-99 and CSW-1 ILTV wild type virus strains were used as parental viruses for *in vivo* co-inoculation. These field strains are classified as class 2 and class 4 viruses [2]. The CSW-1 and V1-99 ILTV genomes have a high level of nucleotide identity (99.8%) [5]. The V1-99 field strain was a predominantly detected in disease outbreaks in Australia until 2008, and was isolated from a hen in a commercial laying flock in 1999 [18]. The CSW-1 strain is commonly used as a standard laboratory strain in Australia and is often propagated in avian cell cultures [19, 20]. It was originally isolated in 1970 from layer birds [21].

### Cell culture and *in vitro* characterization of viruses

Viruses were propagated and characterized in leghorn male hepatoma (LMH) cells. LMH cells are an immortalized chicken hepatocellular carcinoma cell line [22]. Cell monolayers were grown as previously described [19]. Virus titrations were performed to calculate plaque forming units per mL (PFU/mL) as described in Devlin *et al* [23]. *In vitro* multi-step virus growth kinetics and entry kinetics studies were performed as described in Devlin *et al* [23] and Lee *et al* [24], respectively.

### *In vivo* co-infection experiment

*In vivo* studies were conducted with approval from the Faculty of Veterinary and Agricultural Sciences Animal Ethics Committee, The University of Melbourne (Ethics ID 1413401.1) Five specific pathogen free (SPF) chickens, at five weeks of age, were housed together in an isolator unit and provided with feed and water *ad libitum*. Each chicken was co-inoculated by the intra-tracheal route with 300 μL of a mixture of CSW-1 and V1-99 ILTV strains containing 1x10^4^ PFU/mL of each virus. Four days after co-inoculation, at the expected peak of viral replication, [23] all chickens were euthanized by exposure to halothane overdose and tracheal swabs were collected from each chicken into 1 mL of viral transport medium (Dulbecco's Minimal Essential Media (DMEM), 3% v/v foetal bovine serum (FBS) and 100 μg/mL ampicillin). The swabs were transported on ice until they were stored at -80°C for further virus isolation and plaque purification.

### Virus isolation and plaque purification

Each tracheal swab obtained from the *in vivo* co-infection study was serially diluted (ten-fold) in growth media and used to inoculate sub-confluent (80–90% confluency) LMH monolayers in 6-well plates. After 1 hour of adsorption at 37°C, the monolayer was covered with methyl-cellulose overlay media (1% w/v methyl-cellulose in DMEM, with 10% FBS, 50 μg/mL ampicillin, 50 μg/mL gentamicin) and incubated at 37°C. After 24 to 48 hours of incubation, 20 isolated plaques from each swab sample were carefully picked using a micropipette and an inverted light microscope. Then each plaque was individually propagated by inoculating of LMH monolayers grown in 12-well plates. Three plaque picking purification rounds, with one freeze/thaw cycle in-between each round, was performed before a final amplification in one well of a 6-well plate. Nucleic acid was extracted from 200 μL of plaque-purified virus using the automated QIAxtractor (Qiagen) system and Vx reagents (Qiagen). DNA was eluted in 100 μL of elution buffer. In order to control for any recombination that could occur *in vitro* during the process of inoculation and plaque purification, pure CSW-1 and V1-99 stocks were mixed and immediately used to co-inoculate additional cultures of LMH cells using 1:1 ratio of 1×10^4^ PFU of each virus. Isolated plaques were then picked and processed as described above.

### SNP genotyping assay

**Fig 1:**
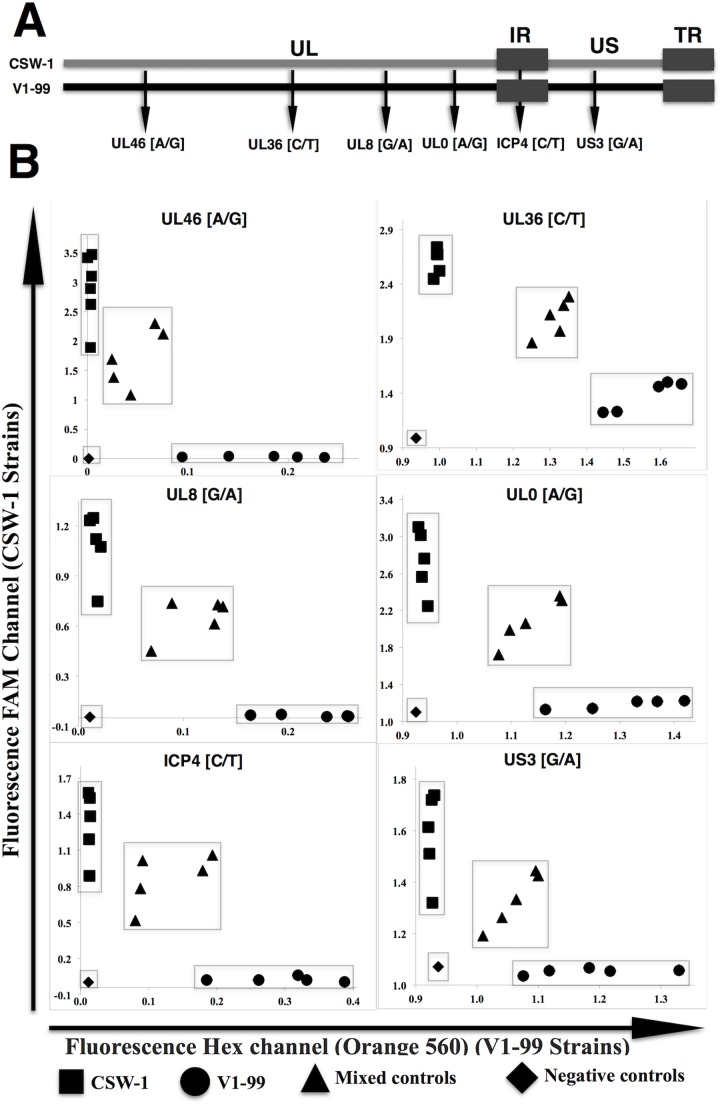
Single nucleotide polymorphism (SNP) genotyping assay. **A)** Schematic representation of the distribution of the TaqMan probes along the unique long (UL), unique short (US), internal and terminal repeat (IR–TR) regions of the genome of CSW-1 ILTV (grey) and V1-99 ILTV (black). The SNPs targeted are indicated with arrows and were located in the UL46, UL36, UL8, UL0, ICP4 and US3 genes **B)** Scatter plot results from SNP genotyping assays applied to three 10-fold dilutions of positive control starting from 1x10^5^ genome copy numbers per reaction (pure CSW-1 (*y* axis as ■) or V1-99 (*x* axis as ●) ILTV DNA), negative controls (no template and DNA negative extractions as ◆) and mixed controls (CSW-1 and V1-99 ILTV genome copy numbers in ratios of 10:1, 1:1 and 1:10 as ▲) obtained from the SNP genotyping assay platform within the Stratagene Mx3000P QPCR 4.1v software with measurements of fluorescence for Orange 560 (*x*-axis) and FAM (*y*-axis) dyes labeling V1-99 or CSW-1, respectively.

To detect recombination a TaqMan SNP genotyping assay was developed that targeted six unique SNPs distributed along the ILTV genomes. The SNP were separated by a maximum of 30 kilobase pairs (kbp), and a minimum of 2 kbp and were selected following alignment of whole genome sequences of CSW-1 and V1-99 ILTV (Genbank accession JX646899 and JX646898, respectively) using the progressive Mauve algorithm in Genious 8.0 software [25] (Fig 1A). All targeted SNPs were transition changes, and they further result in non-synonymous and synonymous changes (Table 1). Each of the six SNPs identified from the full genome sequences of the parental strains were reconfirmed by PCR (primers are shown in S1 Table) and amplicon sequencing (Big Dye Terminator v3.1, Life Technologies). Samples were processed by the Centre for Translational Pathology, University of Melbourne followed by sequence analysis using Geneious software version 8.0 [25]. After the presence of each SNP was confirmed, the target sequences for each SNP were submitted to RealTimeDesign software, Bioserch Technologies (https://www.biosearchtech.com/realtimedesign) (S2 Table) to design appropriate primers and TaqMan probes. Dyes 6-carboxyfluorescein (FAM) and 560 CAL Fluor Orange were selected for all the CSW-1 and V1-99 SNPs, respectively (Table 2). *In silico* examination of the probes and primers was performed using the oligo evaluator feature within the RealTimeDesign software (https://www.biosearchtech.com/ProbeITy/design/OligoEvaluator.aspx).

**Table 1:** Single nucleotide polymorphisms (SNPs) selected within the CSW and V1-99 genomes.

Target gene	Target length sequenced (bp)	SNP position within the gene	Codon modification in bold			Modification type (NS/S)*	Expression kinetics (Early-Late)/Gene related function
			CSW-1	V1-99			
UL46	111	808	[**A**GA] Arg (R)		[**G**GA] Gly (G)	Transition NS	Early-Late/Gene regulation
UL36	88	7703	[G**C**C] Gly (G)		[G**T**C] Asp (D)	Transition NS	Late/Gene regulation
UL8	99	536	[A**G**T] Ser (S)		[A**A**T] Asn (N)	Transition NS	Early/(Helicase-primase,nuclear genes)
UL0	151	1099	[TG**A**] Ser (S)		[TG**G**] Pro (P)	Transition NS	Late/Virulence
ICP4	78	1548	[**C**GG] Pro (P)		[**T**GG] Pro (P)	Transition S	Immediate-early (Gene regulation)
US3	100	207	[GA**G**] Glu (E)		[GA**A**] Glu (E)	Transition S	Early/(Helicase-primase,nuclear genes)

*NS = non-synonymous, S = synonymous

**Table 2:** Oligonucleotide probes and primers used to target single nucleotide polymorphisms (SNPS) within the CSW-1 and V1-99 ILTV genomes

Target gene	SNP position in CSW-1	SNP position in V1-99	Nucleotide identity		Fluorogenic probes (5’—BHQ-1 plus 3’)	Primers (5’ - 3’)
			CSW-1	V1-99		
UL46	21292	22426	**A**	**G**	FAM- AGCCGAAGCTC**T**CCTATT	F:CGTTTCGCGTGTATCCATAAATTCC
					CAL-CCGAAGCTC**C**CCTATTA	R:GCAAACTGGCGTCCTTATTTGAC
UL36	56693	55824	**C**	**T**	FAM-CGGG**C**CCTCGTGT	F:GCGGTGTCATGTTTATCTCTGTG
					CAL-TGGCGGG**T**CCTCG	R:GAAGCCACCTGGCAACAAC
UL8	97644	98771	**G**	**A**	FAM-CGTTCAATATAA**C**TTGCTT	F:GTTCACAACAATGTCCGGCTTG
					CAL-TGCGTTCAATATAA**T**TTGCT	R:CACGTATGGCAACTGCATCTTAC
UL0	108555	109683	**A**	**G**	FAM-TACGGCC**T**CAGACCT	F:GCACGTCCGTCCTCTATACC
					CAL-CTACGGCC**C**CAGAC	R:TTTGCCCTGCGCCATCAT
ICP4	116606	118188	**C**	**T**	FAM-TGTATTTCC**G**GGAGCG	F:ACCTGTTGGCGGCTCTTAG
					CAL-TTGTATTTCC**A**GGAGCGG	R:CAGACGCCGCCGTAGGAT
US3	126141	127700	**G**	**A**	FAM-CGCGGA**G**CCTGTGA	F:CTCGCCGAACTTGTTAGTGTGA
					CAL-CGCGGA**A**CCTGTGAC	R:TCTGCCCGTTCTCGTTAGC

For the TaqMan PCR assay, DNA extracted from pure laboratory stocks of CSW-1 and V1-99 ILTV were used as control samples using three 10-fold dilutions starting from 1x10^5^ genome copy number per reaction (Fig 1B). This extracted DNA was also used to create control samples that contained a mixture of both virus genomes at a ratio of 10:1, 1:1 and 1:10 genome copy numbers. The genome copy numbers were quantified by using a q-PCR targeting the UL15 gene described elsewhere [26]. These pure and mixture controls were then used as template in every qPCR, to enable each SNP to be called using the analysis platform within the Stratagene Mx3000p software. Negative (no template) and negative DNA extraction control reactions that used distilled water as template and sample, respectively, were also included in each qPCR.

Each PCR utilised 2 μL of DNA template, 500 nM of each of the relevant primers and 500 nM of the relevant probe (Table 2), in addition to 8 μL of the TaqManGTXpress Master Mix (Applied Biosystems). All reactions were prepared using the automated QIAgility system (Qiagen). Reactions were performed in the Mx3000 real time thermocycler (Stratagene) and incubated through 1 cycle of 95°C for 2 minutes, 40 cycles of 30 seconds at 95°C and 60°C for one minute. Endpoint readings of the fluorescence from 6-carboxyfluorescein-FAM dye (CSW-1 SNPs), and CAL Fluor Orange 560 dye (V1-99 SNPs) generated during the PCR amplification, were read and plotted using Stratagene Mx3000P QPCR 4.1v software. The results were used to confirm the presence of DNA from either CSW-1 or V1-99 ILTV at each SNP locus (Fig 1). Following validation of the assay using the control samples, the assay was then applied to DNA extracted from plaque purified viruses recovered from the *in vivo* co-inoculation, as well as plaque purified viruses arising from *in vitro* mixing of CSW-1 and V1-99 ILTV, in order to detect and discriminate between parent and recombinant viruses. Any samples that produced a result that could not be definitively identified as either CSW-1 or V1-99 ILTV at any SNP loci (i.e. potentially a mixed infection) were excluded from further analysis.

## Results

### CSW-1 and V1-99 ILTV strains showed similar growth kinetics and entry kinetics in LMH cells

**Fig 2:**
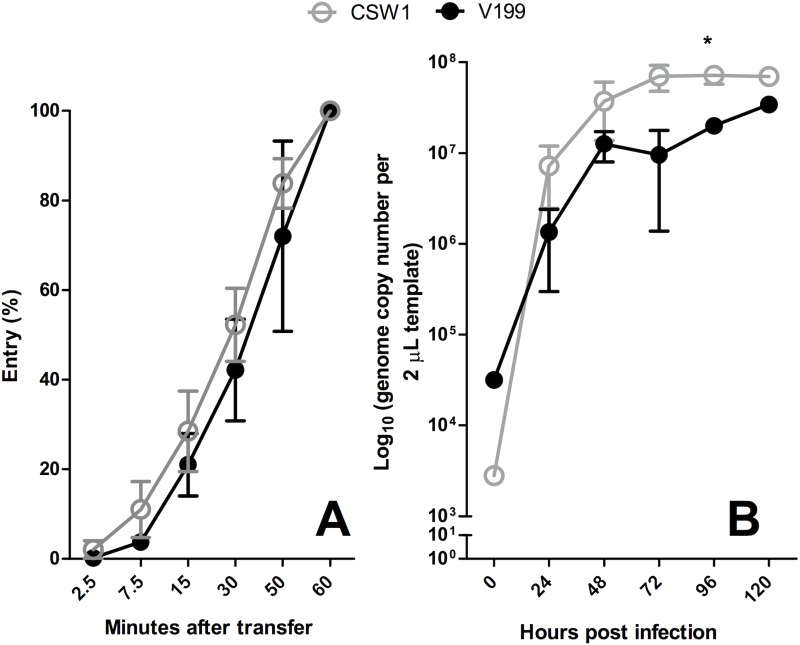
Growth and entry kinetic for the parent viruses, CSW-1 and V1-99 ILTV. **A)** Entry kinetics of CSW-1 and V1-99. LMH cells were infected with virus, after which they were overlaid with methylcellulose medium and incubated at 37°C. Entry at different time points was calculated by comparing the number of plaques formed after inoculation as percentage of plaques formed after an inoculation period of 60 minutes. This experiment was performed 3 times independently. Mean results are shown. Error bars represent the standard deviation. **B)** Growth kinetics of CSW-1 and V1-99. LMH cells were inoculated in triplicate at a multiplicity of infection (MOI) of 0.002. At 24 hours intervals virus genome concentration in the cell-free supernatant was determined by qPCR. Error bars represent standard deviations and asterisks indicate values that were significantly different (*p* < 0.05 Student t test).

Growth and entry kinetics experiments were performed to determine if the CSW-1 and V1-99 ILTV strains showed similar *in vitro* characteristics in LMH cells, and therefore determine the suitability of these strains as parental viruses for the study of ILTV recombination. These experiments were also performed to determine the suitability of LMH cells for the isolation and detection of viral progeny from the collected swabs, without biased recovery of one virus over the other. No significant differences between the entry kinetics of the two viruses were detected (Fig 2A). The multi-step growth curves of the viruses were similar, with only one significant difference detected at 96 hours after infection (Fig 2B). At this time point CSW-1 ILTV had a significantly higher titre than V1-99 ILTV.

### A high proportion of ILTV recombinants were detected following co-inoculation of SPF chickens with CSW-1 and V1-99 ILTV strains

**Fig 3:**
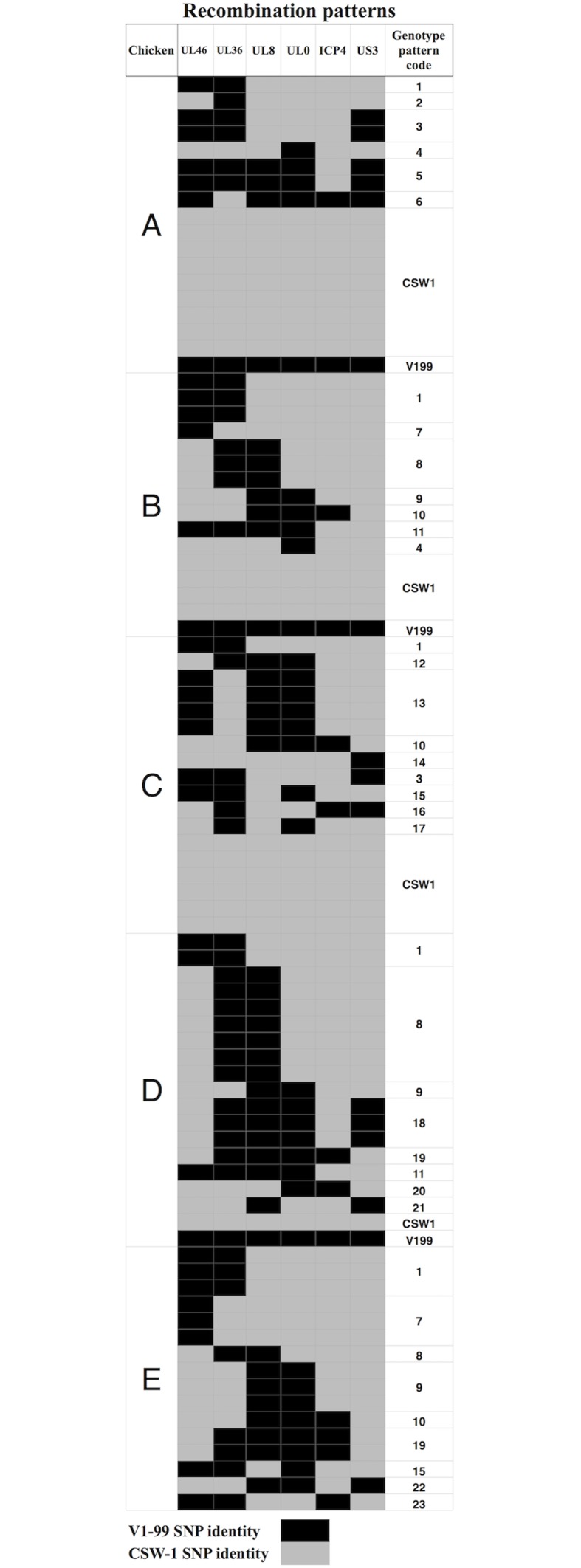
Progeny viruses detected four days after co-inoculation of SPF chickens with CSW-1 and V1-99 ILTV. Results from Taqman SNP genotying assays are shown for 87 viruses isolated and plaque purified from five SPF chickens (A to E) co-inoculated with CSW-1 and V1-99 ILTV. Each row corresponds to an isolated virus, with each SNP represented as CSW-1 origin (grey) or V1-99 (black). Each recombination pattern was given a unique genotype code (last column).

The SNP genotyping assay was applied to DNA extracted from 20 plaque purified viruses from each of the five co-inoculated SPF chickens. In total, 13 of the 100 plaques examined were determined to potentially contain a mixed population of viruses. These plaques (between 1–4 plaques per chicken) were excluded from any further analysis. The results from the SNP analysis of the remaining 87 plaques revealed the presence of parent and recombinant viruses in each of the five chickens (Fig 3). In total 23 different patterns of recombination were detected, with some of them appearing more frequently than others, such as patterns 1 and 8 (Fig 3) (Table 3). The number of recombinant viruses (64/87; 74%) was higher than the number of parent viruses (23/87; 26%) detected. No recombinants were detected in the plaque-purified viruses that were isolated following *in vitro* mixing of CSW-1 and V1-99 ILTV (S1 Fig).

**Table 3:** Summary of recombinant and parental virus progeny detected in co-inoculated SPF chickens.

Genotype pattern code	Number of isolates (%)
1	10 (11.5)
2	1 (1.1)
3	3 (3.4)
4	2 (2.3)
5	2 (2.3)
6	1 (1.1)
7	4 (4.6)
8	11 (12.6)
9	5 (5.7)
10	3 (3.4)
11	2 (2.3)
12	1 (1.1)
13	4 (4.6)
14	1 (1.1)
15	2 (2.3)
16	1 (1.1)
17	1 (1.1)
18	3 (3.4)
19	3 (3.4)
20	1 (1.1)
21	1 (1.1)
22	1 (1.1)
23	1 (1.1)
CSW-1	20 (23)
V1-99	3 (3.4)
**TOTAL**	**87 (100)**

## Discussion

Previous studies have shown that SNP genotyping assays can be used to detect *in vitro* recombination between two closely related strains of the alphaherpesvirus bovine herpesvirus 1 (BHV-1) [27] but this is the first to apply this technique to the study of recombination in ILTV, and the first study to use this technique to study alphaherpesvirus recombination in the natural host. As herpesviruses have a long history of virus-host coevolution extending over 200 million years, studies performed in the natural host can reveal aspects of alphaherpesvirus biology that may not be apparent in studies that utilize laboratory animal models of infection [28]. The results from this study show that the TaqMan SNP genotyping assay is a suitable tool for studying *in vivo* recombination between ILTV field strains in an experimental setting. The SNP genotyping assay, combined with the many advantages of performing infection studies in chickens, over studies in larger animals such as cattle, pigs or horses, demonstrates the suitability of this system as a platform for studying alphaherpesvirus recombination.

Even though the full genomes of CSW-1 and V1-99 share 99.8% sequence identity [5], it was possible to identify six suitable SNPs spread across the genome, out of a total of 73 SNPs present within coding regions, that could be used to differentiate viral recombinant progeny by determining if the target region was from CSW-1 or V1-99 (Fig 1A). Four of these SNPs were located in the unique long (UL) region (UL46-UL36-UL8 and UL0), one in the repeat region (ICP4) and one in the unique short (US) region of the genome (US3). Previous studies carried out in our laboratory have shown ILTV recombination events within the UL and IR region [5] and within the US region [6]. Results from this study indicate that ILTV recombination does not appear to occur in any specific location within the genome, but rather recombination events appeared to result in many different patterns of mosaic viruses. Although some patterns of recombination were detected more frequently that others, it is unclear if this is due to these particular combinations of recombination events occurring independently multiple times, or the detection of multiple progeny recombinants arising from a single recombination event, or a combination of both. Further work to investigate this is indicated, including to assess if specific recombinants may have fitness advantages over other viruses *in vivo*, and if recombinants are horizontally transmitted between birds.

The *in vitro* growth and entry characteristics of the two parental viruses were assessed in LMH cells before the *in vivo* experiment for two reasons. Firstly, to determine if the rate of viral replication and entry of these strains were synchronized, since these two aspects have been identified as important for facilitating recombination [15]. Secondly, to assess the suitability of LMH cells for the isolation of viruses recovered from the *in vivo* experiment. In this regard, the CSW-1 ILTV showed an advantage in replication (higher genome copy numbers 96 hours post-inoculation) compared with V1-99 (Fig 2B). These results are not unexpected given that CSW-1 ILTV is a field strain that is now commonly used in laboratory settings [19, 29] and is therefore more likely to be adapted to cell culture than V1-99 ILTV. Nonetheless, an advantage in replication of CSW-1 ILTV at 96 hours post-inoculation was not considered to be a major disadvantage as plaque purification was performed between 24 and 48 hours post-inoculation. It is interesting to note that the SNP profile consistent with the CSW-1 ILTV parent strain was recovered more frequently from co-inoculated chickens than V1-99 ILTV, and was the only plaque type isolated after *in vitro* mixing of parent strains, suggesting even small advantages in growth potential may be able to influence the recombination potential in infections systems, and this remains for further study.

The SNP genotyping assay described in this study is a powerful tool to study ILTV recombination, and although this technique cannot be expected to have the precision and depth of full genome sequencing techniques, given that it can only detect recombination events within the target genes, the advantages in cost (approximately 1/10 the cost of full genome sequencing), time and ability to apply the assay to large numbers of samples shows that this tool is appropriate for studying ILTV recombination in experimental conditions. Future potential applications of this assay include studies examining recombination under different co-inoculation conditions (e.g. different doses of virus and different times between inoculation of each virus) and also studies to assess how ILTV vaccines may be used to limit recombination. Although ILTV vaccines help to prevent disease they do not fully prevent infection or viral replication following challenge [30]. As alphaherpesvirus recombination is closely associated with replication [15] this means there is the potential for ILTV recombination to occur in vaccinated birds. Different vaccines and vaccination programs are likely to impact recombination differently and this can be examined using ILTV vaccination and challenge (co-inoculation) studies, along with the tools developed in this study. Such future studies are likely to contribute to improved control of disease due to ILTV, and also contribute to a fundamental understanding of alphaherpesvirus recombination.

## Supporting information

S1 Table Primers used for confirmation of SNPs in the CSW-1 and V1-99 ILTV genomes. (DOCX)

S2 Table Sequences submitted to the RealTimeDesign™ software for primer and probe design. (DOCX)

S1 Fig In vitro co-inoculation of CSW-1 and V1-99 in LMH cell culture. (PPTX)
