# Supplementary material for: Development and application of a TaqMan single nucleotide polymorphism genotyping assay to study infectious laryngotracheitis virus recombination in the natural host
Source: PLoS One. 2017 Mar 28;12(3):e0174590. doi: 10.1371/journal.pone.0174590 (PMC5370143; doi:10.1371/journal.pone.0174590)
Supplement: S1 Table — (DOCX) [file pone.0174590.s001.docx]

|  | |  |  |  |
| --- | --- | --- | --- | --- |
| **Supporting information 1.** Primers used for confirmation of SNPs in the CSW-1 and V1-99 ILTV genomes | | | | |
| **Target region** | **Primer sequence (5’ - 3’)** | | **Product length (bp)** | **Annealing temperature (*C)** |
| **UL46** | F:TTGCCCGAATGCTCAGACAT | | **600** | **65** |
|  | R:CCGAAATGAGCTCCCACCTA | |  |  |
| **UL36** | F:GACTTCCTTGTCCCACGTGT | | **796** | **65** |
|  | R:ACATCCAAAGCACTCGAGCA | |  |  |
| **UL8** | F:AGAAAGCGACCTCAGTGTGG | | **599** | **65** |
|  | R:TTTTCGCGGGTGTTAAGGCA | |  |  |
| **UL0** | F:TACGTCGACGAGCAGATGGA | | **639** | **60** |
|  | R:TACCGGAGACAGAAGAGGG | |  |  |
| **ICP4** | F:GCTTGATACAGATCCGGGCG | | **648** | **60** |
|  | R:TCCGAAATCAGCTTCCGTGT | |  |  |
| **US3** | F:TGCAGAAATTTTGCCGACCG | | **450** | **65** |
|  | R:GCCGCCCTTGTTCCATTTTT | |  |  |
