# Supplementary material for: Development and application of a TaqMan single nucleotide polymorphism genotyping assay to study infectious laryngotracheitis virus recombination in the natural host
Source: PLoS One. 2017 Mar 28;12(3):e0174590. doi: 10.1371/journal.pone.0174590 (PMC5370143; doi:10.1371/journal.pone.0174590)
Supplement: S2 Table — (DOCX) [file pone.0174590.s002.docx]

**Supporting information 2:** Sequences submitted to the RealTimeDesign™ software for primer and probe design

| Targeted gene | Sequence submitted | SNP identity [CSW-1/V1-99] |
| --- | --- | --- |
| **UL46**  **UL36**  **UL8**  **UL0**  **ICP4**  **US3** | >UL46 GCACCTTTAAGTTCATTGAAGGATACGCTTATTATATGCGCCCCCCTGGGCCAGAACTCCATTCAACAGAAACGACCGCTCGTCTAGCAGCCCTCCTTGTATATGTGAGAAGCATGTACTGCAAACTGGCGTCCTTATTTGACGCAATCGATGCCGCTGTGCTGCGCTCTAAATTTAATAGG[**A/G]**GAGCTTCGGCTCAAGCTCTGACTGGAATTTATGGATACACGCGAAACGGGAAGGGAACTATAATGTCGCGGTATTCTTCGGAGGCCATGAGTGATGAAATCATGTCTGTGTGTCGCACTGGTTCGGACACTTTGATAGAATCTTTACGCAAAGCTCTGGGGAGATTGACCTACCTTATTGCTAGGTGGGAGCTCATTTCGGTAGAGAAG  >UL36 CTCTGCGAGAAGATTCCTCGTGAAGTTTGTGCGACCCTTTGACATATGCCGGTGTAAAATCCTCGTCAGAACTTTCTCCTTCCGGGGGATCGACTTCCTTGTCCCACGTGTAACTGTATGTTGGGAAGTGAGCTATATCCTCATTATCTGAGCAATGTGTCTGTTTGGCTTCCGCGGTGTCATGTTTATCTCTGTGGCTTGTTATGCTTTCTGGCGGG[**C/T]**CCTCGTGTTCGCTAGTTTTACGTGTTGTTGCCAGGTGGCTTCGATCTTCTGGTTCCGGGTCTGTGGGATGCTCAAGGTTAGTCTCGTCTGTAATAACTGCGAAATTATCAGACGGGTCAGAAGTTATTGACGGACTCTGAATTTTCCAATCACAAGTTTCTGAGA  >UL8  CGCGCCTTGACTCTGCCAGAATTTTATTCTCCTTCTATTTTTTATGTTTGCAAAGACAGTGGATTGATCACTAAGGTCTGCGAGGATAAATCCAAGCCACGTATGGCAACTGCATCTTACGCAGCATTGAATGCTTCGTCTAACTTCCAAGCAA[**G/A]**TTATATTGAACGCAATCTGCAAGCCGGACATTGTTGTGAACTGAGAAATTTTGGATGGGCTCGCATACAGGCAATCTCATCTGGAAAAATTAATCCAAGAAGCATGACCGCGGAATGGGTGTGGGCTGGTGGAAAGTGGATTGATGGAAGAGGAAATGAAGCATTTTC  >UL0  CGTATTTTGCCCTGCGCCATCATAATTAGACGAGGGGTGGTGGGGCGAAAGCTGTGGAGGGGAGCGTCTAGATGGCGAGGGGAGGGATGATAACCTGAGGTCTG**[A/G]**GGCCGTAGGAGATGGCGCAGAAAATAAAGGGGGTATAGAGGACGGACGTGCGGGCGGTGGACTATGAGCGGGGCTATTTTCTATTTCATGCCGCACGACCCGTCGCACCCACGACATCTCATATACATCTCGCACTTCTGCTCTACTCGCAAGGCCATAGTTGCCTTCCCCCTCTTCTGTCTCCGGTAACATAAACGTATCGAATACGGT  >ICP4  GGTTGCCGCAGGTGGCTCTTTCGCTGCGGCTTCCAGCTCCCTCGTGGCACGCGCTATTCGCCTGGCGCCAACGAGTACAGGGTGGTCCCCAGAAGCAGACGCCGCCGTAGGATCCACGGCCGCTCC**[C/T]**GGAAATACAAACGGGACAAACGCTCTCCTAAGAGCCGCCAACAGGTAAGTTTTCTGCGGGTGGTCATAGCGGCGGCACACGCGTACGAGCTCGGTAAGGTGCGGCAGAGCTAATGACACACGGCCATCACGCATGGCGTGCGCTACGTGCGGGAGGCACGTCGGCACCGCAGAACGAGACAATCCTCCCTCGG  ACAACCGAGAATCCGCTTACCTCAAAACGCGTTTGCGTATTGGATAGTTTCTCACGGACAATGTCATTGCGCCCCTATGCAGAAATTTTGCCGACCGCGGAAGGCGTCGAGCGCCTCGCCGAACTTGTTAGTGTGACAATGACAGAACGCGCGGA**[G/A]**CCTGTGACAGAGAATACAGCTGTAAACAGTATTCCCCCGGCTAACGAGAACGGGCAGAACTTTGCATCTGCAGGCGATGGGGCCTCGGCTACTGAAAAAGTTGACGGCTCGCATACAGACTTCGATGAAGCATCGAGCGACTACGCCGACCCTGTCCCGCTCGCGCAAACTAGATTGAAGCATTCGGATGAATTTCTTC | **[A/G]**  **[C/T]**  **[G/A]**  **[A/G]**  **[C/T]**  **[G/A]** |
