# Supplementary material for: Development and application of a TaqMan single nucleotide polymorphism genotyping assay to study infectious laryngotracheitis virus recombination in the natural host
Source: PLoS One. 2017 Mar 28;12(3):e0174590. doi: 10.1371/journal.pone.0174590 (PMC5370143; doi:10.1371/journal.pone.0174590)
Supplement: S1 Fig — (PPTX) [file pone.0174590.s003.pptx]

## Slide 1
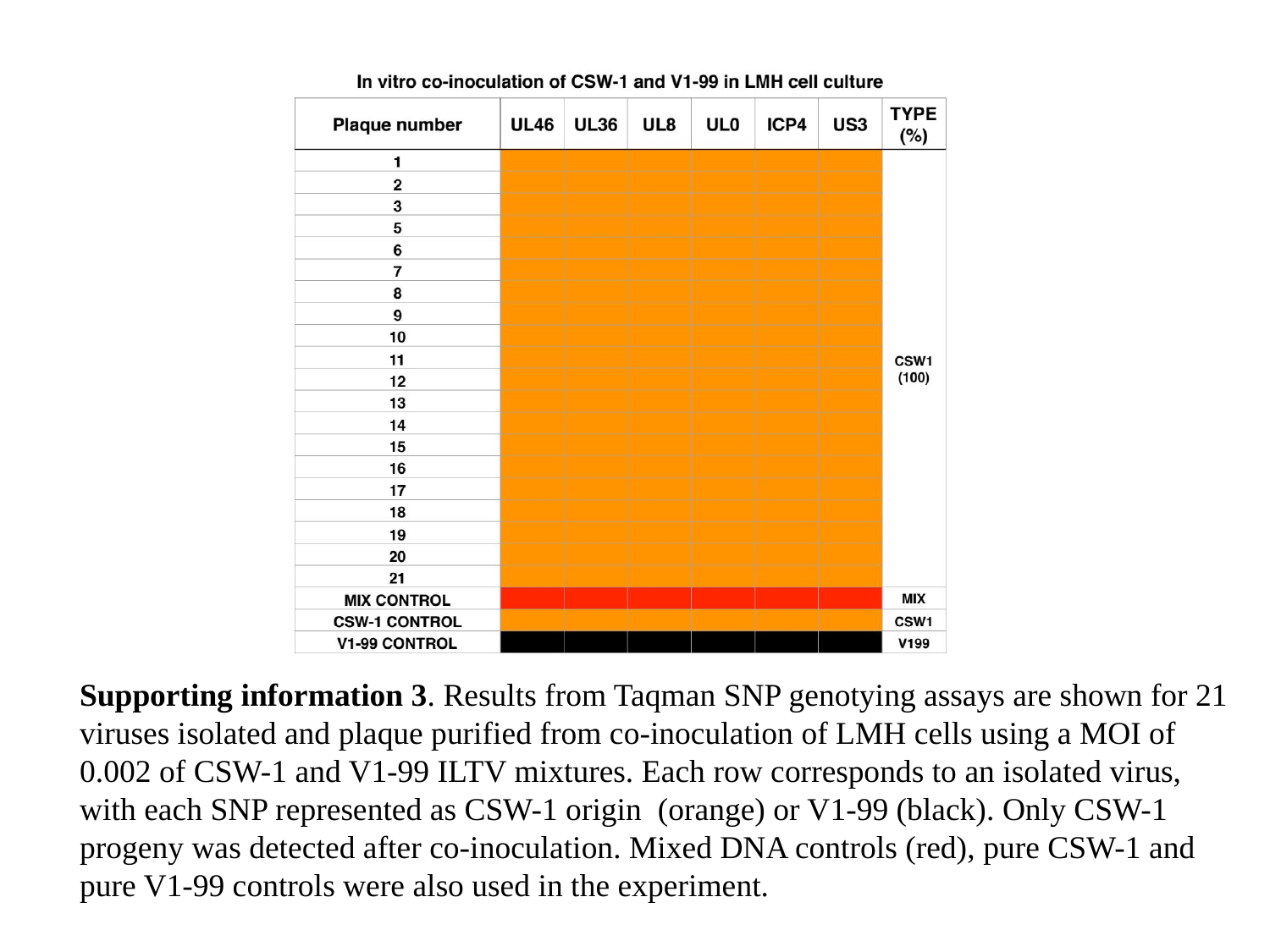

Supporting information 3. Results from Taqman SNP genotying assays are shown for 21 viruses isolated and plaque purified from co-inoculation of LMH cells using a MOI of 0.002 of CSW-1 and V1-99 ILTV mixtures. Each row corresponds to an isolated virus, with each SNP represented as CSW-1 origin (orange) or V1-99 (black). Only CSW-1 progeny was detected after co-inoculation. Mixed DNA controls (red), pure CSW-1 and pure V1-99 controls were also used in the experiment.
